# How Far Could We Go with Open Data – A Case Study for TRPV1 Antagonists

**DOI:** 10.1002/minf.201300019

**Published:** 2013-06-18

**Authors:** Daria A Tsareva, Gerhard F Ecker

**Affiliations:** [a] Department of Medicinal Chemistry, University of ViennaAlthanstr. 14, 1090 Vienna, Austria phone: +43-1-4277-55110

**Keywords:** Open data, TRPV1, GRIND, Classification

## Abstract

Publicly open databases of small compounds have become an indispensable tool for chemoinformaticians for collection and preparation of datasets suitable for drug discovery questions. Since these databases comprise compounds coming from structure-activity relationship (SAR) studies performed by different research groups, they are very diverse with respect to the biological assays used. In the present study we analyzed the applicability of a thoroughly curated dataset gathered from open sources for ligand-based studies, using the transient receptor potential vanilloid type 1 (TRPV1) as use case. Thorough curation of compounds according to the biological assay type and conditions led to a dataset of comparable bioactive chemicals. Subsequent exhaustive analysis of the obtained dataset using classification algorithms demonstrated that the models obtained in most of the cases possess reliable quality. Analysis of constantly misclassified compounds showed that they belong to local SAR series, where small changes in structure lead to different class labels. These small structural differences could not be captured by the classification algorithms. However application of the 3D alignment-independent QSAR technique GRIND for local, structurally related series overcomes this problem.

## 1 Introduction

With the availability of open access databases, such as ChEMBL DB,^1^ PubChem,^2^ Drugbank,^3^ and IUPHAR,^4^ the pharmacoinformatics community now has access to millions of data points. These data are mostly compiled from literature sources and are thus quite heterogeneous with respect to the biological assays used. Furthermore, these datasets mainly comprise compounds from different local SAR series and therefore show an inhomogeneous distribution both within the biological property space and the chemical space. This renders it difficult to create global QSAR models based on open data.

In this case classification algorithms seem to be the method of choice. They could be an excellent tool for exploration of the diverse chemical space provided in open data sources. Moreover, separation of data points through classification models has proven to be a fast and reliable tool in computational chemistry.^5^,^6^ In an attempt to exploit the wealth of open data available and to check the performance of classification algorithms applied to these data we aimed to collect, systematize and analyze a set of data available in ChEMBL DB. As a model target we chose the transient receptor potential vanilloid type 1 (TRPV1), which became of interest to us in light of our work on piperine-type compounds.

TRPV1 is a transmembrane ion channel and is mainly located in the nociceptive neurons of the peripheral nervous system. It is responsible for the transfer of pain stimuli from periphery to the central nervous system and thus it represents an emerging target for development of analgesics.^7^ Since the crystal structure of the receptor is not available, current in silico work mainly focuses on ligand-based approaches.^8^ Availability, variety and diversity of the information on ligands in CHEMBL DB makes it an interesting use case for exploring the usability of open data.

## 2 Methods

### 2.1 Preparation of the Datasets

The 2D structures of 2332 TRPV1 ligands were downloaded from the ChEMBL DB release 13. Since the ChEMBL DB provides broad and diverse information on type and range of activity and biological assays of the compounds, the derived structures consequently underwent a rigorous filtering protocol. At the beginning, 1479 compounds with one type of measured activity on the TRPV1 receptor (i.e. IC_50_ values) were chosen from the whole list (see Table 1). Next, the dataset was filtered according to the assay type used. For 1479 TRPV1 ligands the ChEMBL DB provided 78 different assay descriptions, the whole list of which can be found in Table SI-1 of the Supporting Information (SI). Further, 374 compounds measured on CHO and 1321N cells were removed. This led to dataset of 1105 compounds in which 609 compounds were determined with the use of several assays on HEK293 cells and for 496 compounds the cell line was not mentioned in the assay description (see Table 2 for details). Subsequently, assay type and cell line was manually rechecked in the literature reference provided by ChEMBL.

**Table 1:** Different activity types provided by ChEMBL13 for TRPV1 ligands and number of compounds for which corresponding activity was measured

Activity type	Number of compounds
*IC*_50_	1479
*K*_i_	146
*EC*_50_	147
Activity	106
Inhibition	61
Other[a]	393

[a] Potency, *E*_max_, Hill coefficient, efficacy, response, p*K*_b_

**Table 2:** The assay descriptions available in CHEMBL13 for TRPV1 ligands and number of compounds measured with the corresponding assay. AA: antagonistic activity

Assay description	HEK293	N/A
AA at hTRPV1 assessed as inhibition of capsaicin-induced calcium influx	406	274
AA at hTRPV1 assessed as inhibition of acid-induced calcium influx	95	32
AA at hTRPV1 assessed as inhibition of capsazepine-induced calcium mobilization	16	–
AA at hTRPV1 assessed as inhibition of capsaicin-induced effect at pH 5.5	3	–
AA at hTRPV1 as decrease in intracellular calcium levels	29	84
AA at hTRPV1 assessed as inhibition of N-arachidonoyl-dopamine-induced effect	1	1
AA at hTRPV1 assessed as inhibition of PMA-induced activation	12	6
AA at hTRPV1 assessed as inhibition of agonist-induced increases in intracellular [Ca^2+^] levels	40	–
Inhibition of anandamide activated hTRPV1 receptor in [Ca^2+^] influx assay	–	13
Inhibition of hTRPV1	7	53
Inhibition of binding to hTRPV1	–	20
hTRPV1 blocker	–	13

Consequently, 531 compounds reported as blocking the capsaicin-induced Ca^2+^ flux in HEK293 cells remained. Finally, the dataset was cleaned from duplicates and from compounds showing unclear activity values, which led to a set of 408 TRPV1 antagonists being comparable between each other. The final dataset thus contained only around 25 % of the compounds reported as TRPV1 antagonists. This once more stresses the need of standardized assays in order to allow optimal use of the wealth of data available, as also outlined recently for another use case.^9^ The range of *IC*_50_ values in the dataset varied from 0.4 to 17490 nM. The activity of capsazepine, a potent antagonist of the TRPV1 receptor measured in this assay type is 100 nM.^10^ This value thus served as threshold to divide the compounds into active and inactive in their ability to block the receptor after its activation induced by capsaicin. This led to a balanced dataset with 201 active and 207 inactive compounds, respectively. A full list of structures together with the *IC*_50_ values and the class labels is provided as sdf-file in the Supporting Information and on our web-page (pharminfo.univie.ac.at).

As descriptors we selected both a set of 2D- and 3D-descriptors. As 2D-descriptors we compiled 32 Van der Waals surface area (VSA)^11^ descriptors implemented in MOE. Each descriptor is computed as the sum of accessible atomic Van der Waals surface areas in a specific range for a given property. The properties usually described are partial atomic charges (PEOE),^12^ molar refractivity (SMR),^11^ and water-octanol partitioning coefficient (*S*log*P*).^11^ For 3D-representation, we chose 76 i3D VolSurf (VSURF) descriptors,^13^ which reflect physico-chemical and pharmaco-kinetic properties in a high dimensional space. The i3D descriptors are calculated based on 3D conformation of each molecule, but are invariant to translations and rotations of the entire conformation. Both VSA and VSURF descriptors have been successfully applied for the analysis of large databases, as well as for QSAR/QSPR analysis.^13^,^14^ Composition of each set of descriptors is given in Table SI-2. The 3D conformations of the compounds for calculation of VSURF descriptors were generated in the Molecular Operation Environment version 2010.10 (MOE)^15^ and minimized using the MMFF94x force field.

The obtained descriptor values for 408 compounds were subjected to *Z*-Score normalization (script is provided in SI) separately for VSA and VSURF descriptor sets. In order to evaluate the robustness of the models obtained, we prepared 100 different collections of training (TR) and test (TS) sets. Each set comprised 80 % (325 compounds) for training and 20 % (83 compounds) for testing. The split was obtained by using different random seeds as implemented in KNIME v.2.3.4.^16^

In addition, so-called “overall” models were built for the dataset without separation on TR and TS.

### 2.2 Classification Algorithms

Machine learning methods used comprise 12 different classification algorithms which are implemented in WEKA.^17^ As decision trees NBTree (Naive-Bayes Decision Tree^18^) and BFTree (Best-first Decision Tree^19^) were chosen. They represent data in a tree-like structure, so that on each node the value of a certain descriptor is taken into account, consequently leading to a splitting of the data. The Random Forest (RF)^20^ algorithm in which a family of trees built from random data subsets and random subsets of features is obtained, was also used in the study because this type of classification is especially useful for fast and robust management of highly variable data. Instance-based learning algorithms used comprised IB1 and IBk.^21^ These similarity searching algorithms evaluate the remoteness of a given new instance from the nearest one or from several neighbors. Probability methods based on Bayesian statistics included Bayesian Logistic Regression (BLR)^5^ and Bayesian Network (BayesNet).^22^ Combination of different outputs into a single prediction (model) was performed using the following: Bagging,^23^ Multiboost (MBoost),^24^,^25^ Dagging,^26^ Decorate^27^ and Ensemble Selection (Ensemble).^28^ In Bagging training data was splitted into several datasets, then a REPTree^17^ was applied to each dataset independently. In MBoost the models for the splitted data were built using Decision stump^29^ and further the vote was assigned to each of the models for combining them into one model. In Dagging the data were divided into several disjoint folds and each basin of data was treated with an SVM (SMO^30^ with polykernel and default parameters as implemented in WEKA). During the Decorate one ensemble of several J48 classifiers were built by constructing special artificial examples for training data. In Ensemble forward selection was used to add the next best tree to an ensemble of REPTrees. For all the above mentioned ensemble techniques the prediction was obtained by averaging the predictions obtained from each model in the ensemble with the exception of MBoost, where the weighted average was computed for final prediction.

### 2.3 3D QSAR. GRID Independent Molecular Descriptor Analysis

3D QSAR analysis was performed using alignment-independent 3D-descriptors in Molecular Discovery software Pentacle version 1.0.6.^31^ The input 3D conformations were generated using the software package Corina v.3.2.^32^ Four types of probes: DRY (descriptor of hydrophobic interaction), O (H-bond acceptor group descriptor), N1 (H-bond donor group descriptor) and TIP (shape descriptor) with a default Grid Step of 0.5 Å were used for computation of Molecular Interaction Fields (MIFs). The discretization of MIFs was performed using the AMANDA algorithm^33^ with default values of probe cutoffs (DRY=−0.5, O=−2.6, N1=−4.2, TIP=−0.75) and a scale factor of 0.55. For MIF encoding, the CLACC algorithm with “Remove non-consistent couples” set to “True” and other parameters to default values was used.

### 2.4 Evaluation of Models

Several standard parameters based on a confusion matrix built on true positives (TP), true negatives (TN), false positives (FP) and false negatives (FN) were used to estimate the quality of the obtained models. Values of sensitivity, specificity, accuracy and Matthews correlation coefficient (MCC)^34^ were obtained for each model applying 10-fold cross-validation of the TR and prediction of TS (see Equations 1–4 in SI).

## 3 Results and Discussion

### 3.1 Overview of the Results – Overall Trends

**Figure 1:**
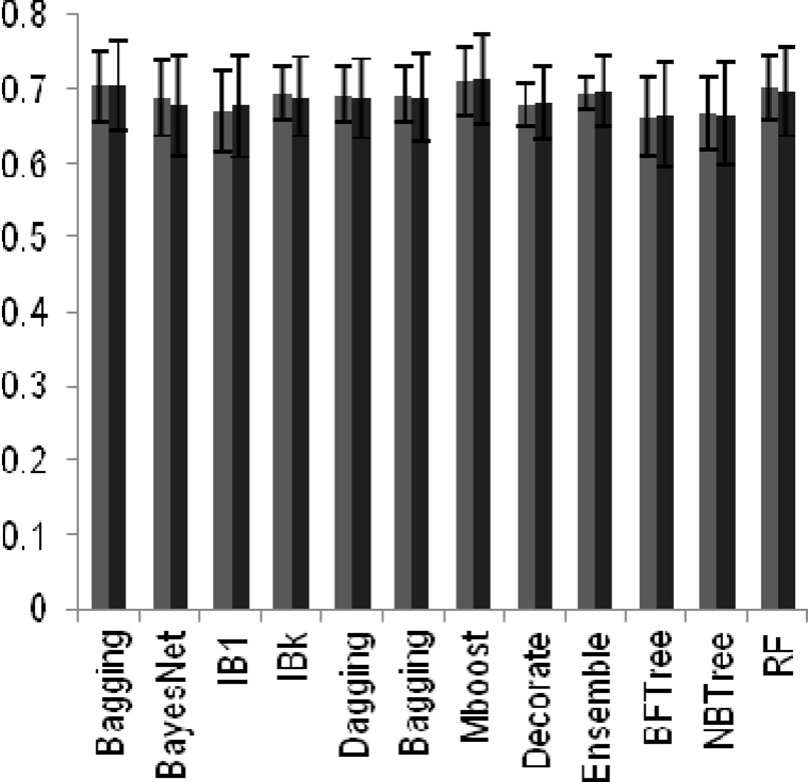
Mean values of accuracy obtained for cross-validation of 100TRs (grey) and prediction of 100 TSs (black) with various classification algorithms. Whiskers stand for standard deviations.

In total, 2400 models for 100 collections of TR and TS sets with 2 descriptor sets and 12 classification methods have been built and their parameters are summarized in Table SI-3. Table SI-4 provides the sensitivity, specificity, accuracy and MCC values for the prediction of the test sets. Generally, the accuracies of the models obtained for our dataset were in the range from 0.5 to 0.9 (see Figure 1).

**Figure 2:**
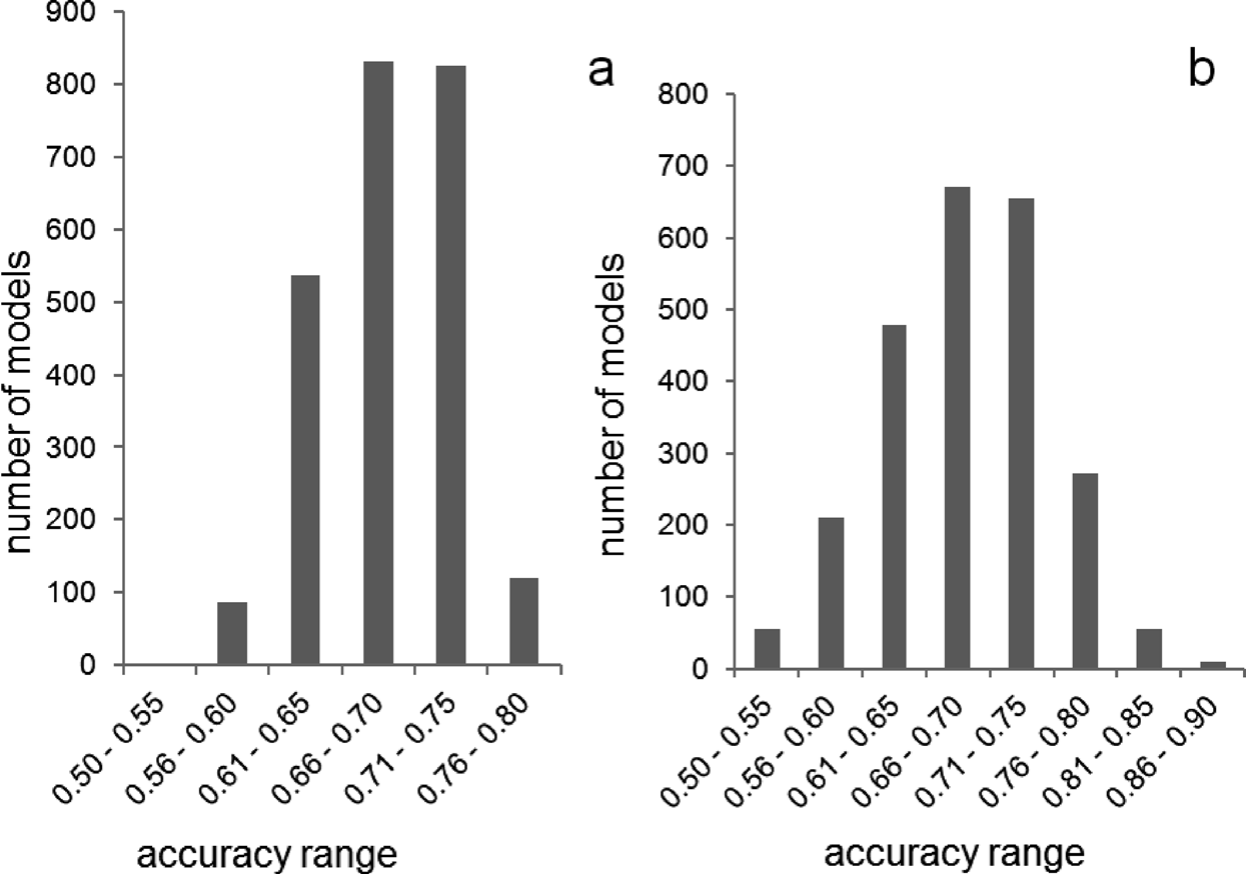
Distribution of values of accuracies a) for 2400 models built with 10-fold cross-validation of training sets; b) for prediction of 100 test sets with every model.

Unfortunately, none of the methods showed consistently high performance (i.e. according to the results no algorithm could be selected with which the models obtained would always show high values of sensitivity and specificity for all the datasets). However, the accuracy shown in cross-validation was at least 0.6 for 98.04 % of the models, 0.7 for 80.04 %, 0.75 for 48.08 % and 0.8 for 9.20 %. The distribution of values of accuracies obtained for 2400 classification models is shown on Figure 2a.

Distribution of values of accuracies for prediction of TS sets based on the corresponding cross-validated models is shown on Figure 2b. The range of accuracies obtained for TS prediction was broader and there were more cases (7.75 % in prediction to 1.96 % in cross-validation) when compounds were almost randomly classified with the model (i.e. total accuracy<0.6). But nevertheless, prediction of TS was higher than 0.7 for 47.75 % of cases compared to 48.08 % for CV models meaning that half of the models built could produce reliable predictions.

**Figure 3:**
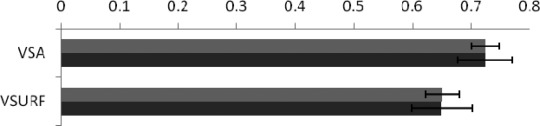
Mean values of accuracy for 100TR-TS sets depending on the descriptor set used for building the model (TR (grey) and TS (black) sets). Whiskers stand for standard deviations.

We have also collected and analyzed the accuracies obtained for the TR and TS sets depending on the descriptor set used. Slightly higher values were obtained in the models based on VSA descriptors both for cross-validation and prediction of TS (see Figure 3). This might be due to the general concept of the VSURF descriptors. They are incremental 3D descriptors (distinguished from VSA, which are 2D), thus the descriptor values depend on the conformation of the studied molecules. Though we have used the energy minimized conformation it might not relate to the bioactive one, which renders it difficult to capture the genuine relation between biological activity and VSURF descriptor values in the models.

### 3.2 Overall Models

Table 3 provides a short overview of the parameters obtained for the overall models. The tendency of VSA descriptors to outperform VSURF descriptors was also observed in the overall models for each classification algorithm. This is exemplified by the values obtained for the MCC, which was always higher than 0.4 in case of VSA descriptors.^35^

**Table 3:** Parameters of the overall models obtained for 12 classification methods with VSA (I) and VSURF (II) descriptor sets

Method	Sensitivity		Specificity		Accuracy		MCC	
				
	I	II	I	II	I	II	I	II
BLR	0.72	0.70	0.71	0.62	0.72	0.66	0.43	0.33
BayesNet	0.78	0.65	0.69	0.61	0.73	0.63	0.47	0.27
IBk	0.73	0.63	0.66	0.67	0.69	0.65	0.38	0.30
IB1	0.73	0.63	0.66	0.67	0.69	0.65	0.38	0.30
Dagging	0.82	0.76	0.66	0.54	0.74	0.65	0.48	0.30
Bagging	0.77	0.66	0.72	0.71	0.74	0.68	0.49	0.36
MBoost	0.91	0.66	0.57	0.51	0.73	0.59	0.50	0.18
Decorate	0.75	0.69	0.70	0.63	0.72	0.66	0.44	0.32
Ensemble	0.78	0.67	0.71	0.71	0.74	0.69	0.48	0.37
BFTree	0.75	0.58	0.68	0.68	0.71	0.63	0.43	0.26
NBTree	0.77	0.67	0.75	0.61	0.76	0.64	0.52	0.28
RF	0.78	0.7	0.71	0.63	0.75	0.66	0.49	0.33

**Figure 4:**
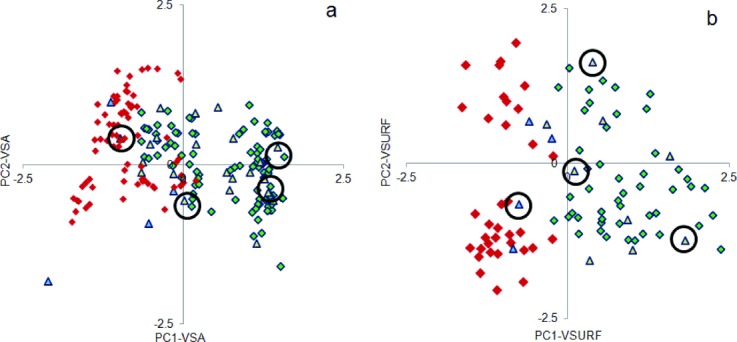
PCA plot of instances which were always predicted as TP, TN, FP and FN by 12 classification methods in VSA (a) and VSURF (b) descriptor space. TP and TN instances are represented as green and red diamonds, FP and FN instances are yellow and cyan triangles, respectively.

Analyzing the models in more detail, we identified 27 and 13 cpds which were constantly wrong classified by every method using the VSA and VSURF descriptor set, respectively. Furthermore, 4 of these compounds were constantly ′misbehaving′ regardless of the classification method and the descriptor set used, i.e. they were constantly misclassified by every overall model obtained. These instances are marked by circles in the PCA plot (Figure 4) and are discussed in detail below.

**Figure 5:**
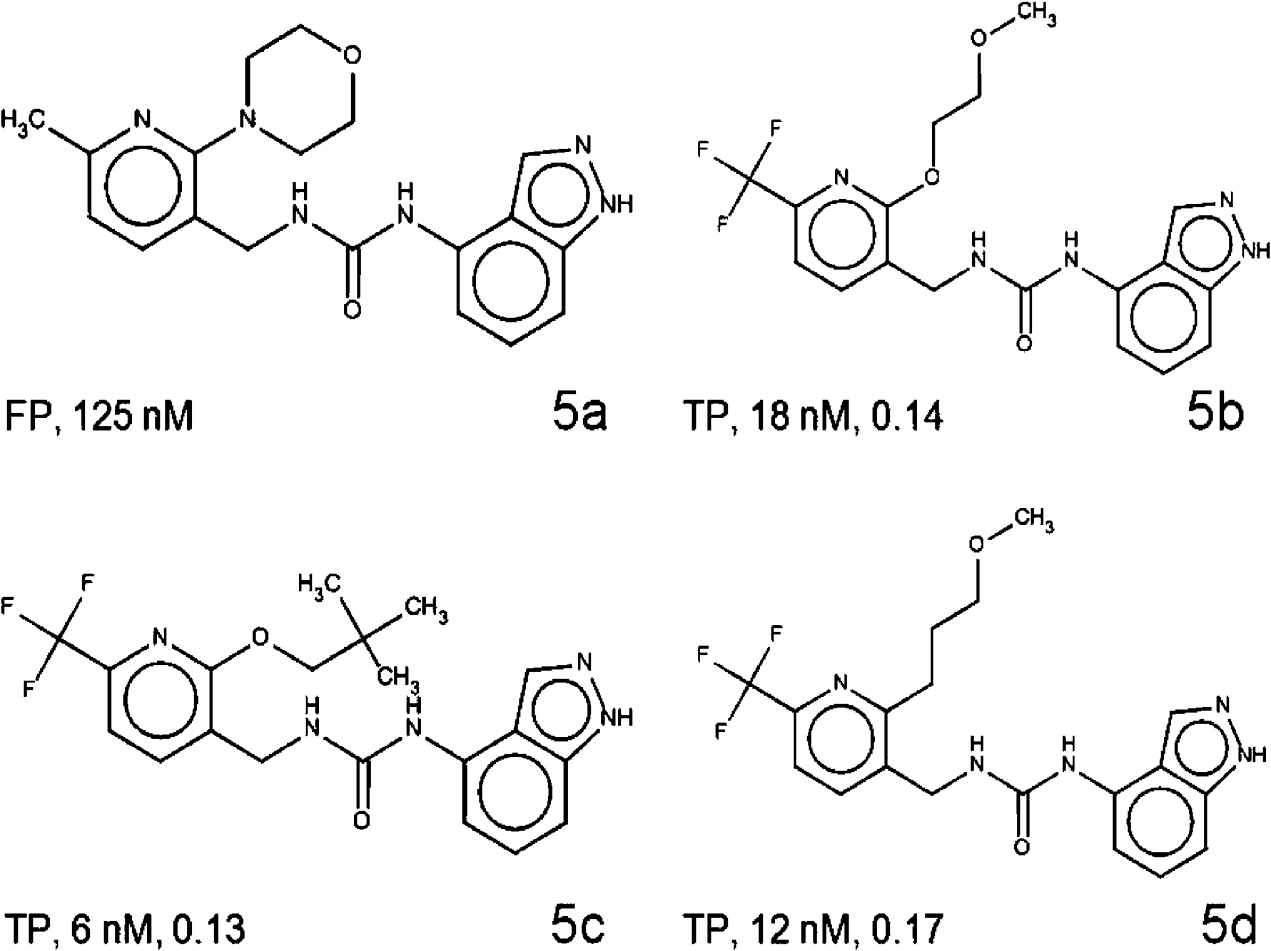
FP-classified compound and its TP-classified nearest neighbors in VSA descriptor space. For each nearest neighbor the Euclidean distance from the misclassified compound in the VSA descriptor space is given.

**Figure 6:**
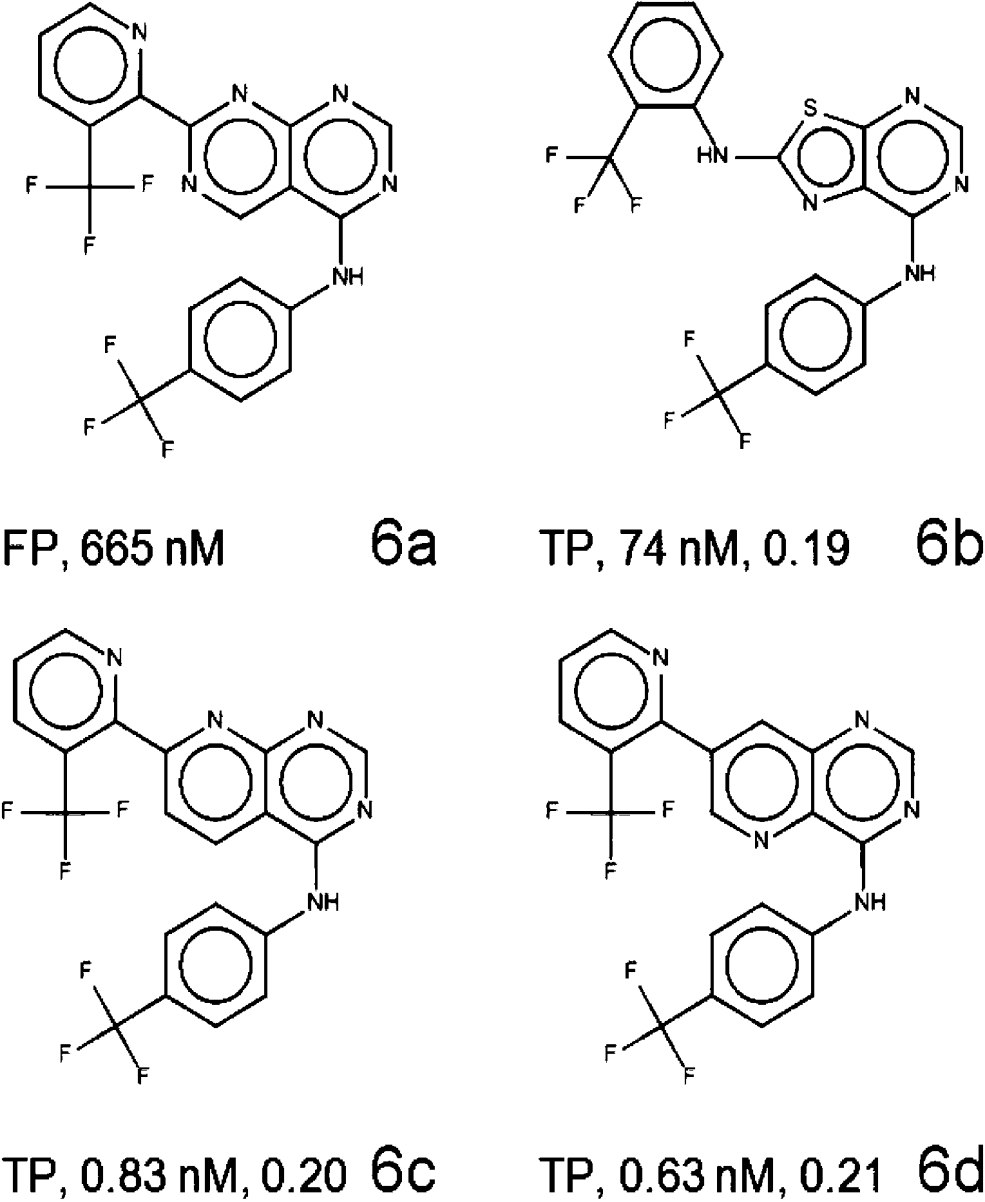
FP-classified compound 5 and its TP-classified nearest neighbors in VSA descriptor space. For each nearest neighbor the Euclidean distance from the misclassified compound in the VSA descriptor space is given.

**Figure 7:**
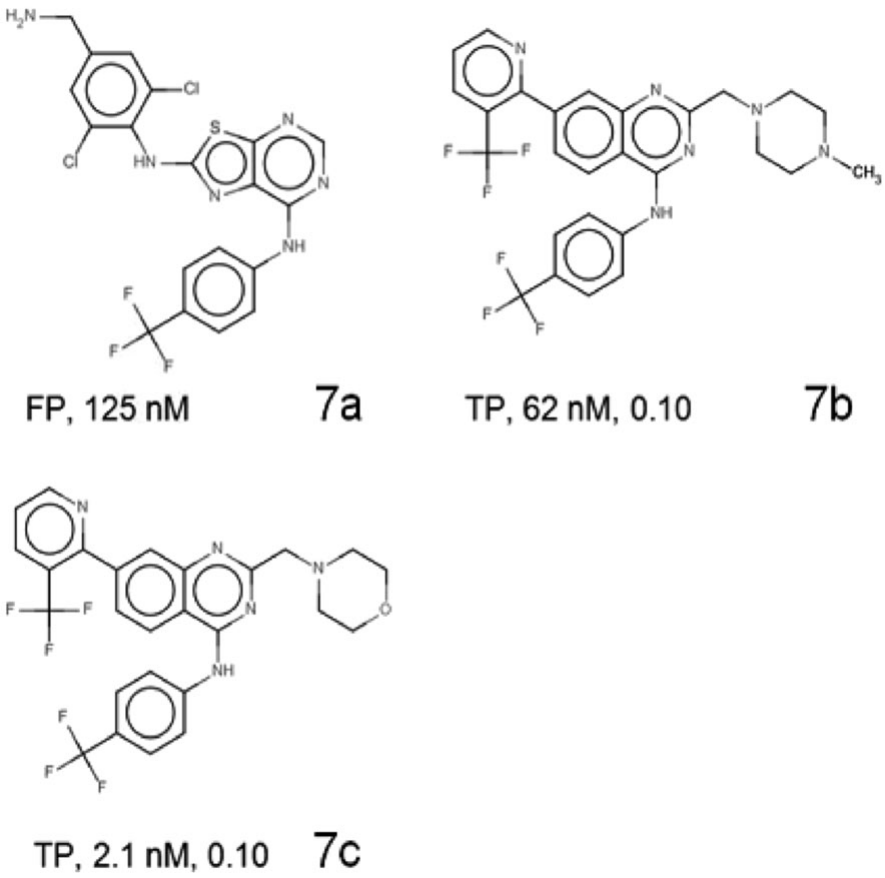
FP-classified compound and its TP-classified nearest neighbors in VSA descriptor space. For each nearest neighbor the Euclidean distance from the misclassified compound in the VSA descriptor space is given.

3 cpds are false positives (FP) and are marked as yellow triangles on Figure 4. Their chemical structures are given in Figures 5a, 6a and 7a. Compound **5a**, as well as its nearest neighbors in the VSA descriptor space (**5b**–**5d** (Figure 5) comprise indazole derivatives developed by Brown et al.^36^ They share the same chemical scaffold and differ solely in the substitution pattern at the pyridine ring. While the FP **5a** shows a methyl- and morpholine-substituent in positions 1 and 5 of the pyridine ring, the TPs **5b**–**5d** exhibit triflouromethyl- and alkyl-substituents. This minor differences in the structure obviously could not be grasped by the classification algorithms because the values of descriptors are very close and therefore the compounds are assigned to the same class (see Table SI-6). A GRIND model built for 20 indazole derivatives (pIC_50_: 8.456–6.735) (sdf-file with the structures is provided in SI) showed a satisfactory 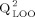 value of 0.52 and an *R*^2^ value of 0.87. However, the first two principal components explained only 19 % of the variance. Nevertheless, the FP compound **5a** could be distinguished from its neighbors. The distance between the H-bond acceptor and the H-bond donor probes (O-N1) of 14.4–14.8 A is shown to be important for the decrease of activity. This distance is present between the NH-group of the indazole moiety (O-probe) and the center of the morpholine ring (nitrogen and oxygen atoms, i.e. N1 probe) in the false positive **5a**, but not in its near neighbors **5b**–**5d**. Alternatively, the *IC*_50_ value of cpd **5a** is with 125 nM very close to the chosen threshold.

Cpd **6a** is an outstanding example of misclassification in our overall models (see Figure 6). Though it is extremely similar to its nearest neighbors in the VSA descriptor space there is 1000-fold difference in activity between cpds **6a** and **6d**. According to the corresponding SAR studies^37^,^38^ great improvement of potency of TRPV1 antagonists is achieved through introduction of an acceptor N into positions 5 and 8 of the central quinazoline ring. This is also in accordance with the relative high potency of cpd **6b**, which has S and N in these positions.^38^ Therefore, introduction of the N into the 6th position of quinazoline presumably should not cost such a dramatic loss in activity. It could not be captured by classification methods since descriptor values are very similar for these four cpds (see SI-6). We supposed that interaction of free electron pairs of pyridine N and N in the 6th position of quinazoline ring of compound **6a** could be unfavorable and cause stabilization of a conformation different from cpds **6b**–**6d**. It would lead to different active conformation of cpd **6a** and therefore loss of valuable interactions with the target and drop of potency compared to its neighbors. To further elaborate this, we performed 3D QSAR studies for these compounds. A GRIND model was built for a dataset of 54 quinazoline derivatives comprised from compounds used in the original SAR study^37^ (sdf-file with the structures is provided in SI). The three-latent variable model had an *R*^2^ of 0.84 (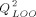 of 0.50). The model was not excellent but it could distinguish compound **6a** from its neighbors **6c** and **6d**. Distances of 1.6–2 Å between shape probes (TIP-TIP) and of 11.2–11.6 Å between H-bond acceptor and shape probes (O-TIP) were shown to be important for the increase of activity and are present in compound **6d**. In contrast, distances of 9.2–9.6 Å between hydrophobic and shape probes (DRY-TIP) and of 6–6.4 Å between hydrophobic and H-bond acceptor probes (DRY-O) responsible for the decrease in activity are present only in FP compound **6a**. The interaction between free electron pairs of nitrogens in pyridine and quinazoline rings, namely distances between H-bond acceptor probes, was not shown to influence the activity. Nevertheless, conformation of compound **6a** was different from those of compounds **6c** and **6d** and different distribution of N1 molecular interaction fields for these structures was observed. This demonstrates that the activity cliffs observed can well be captured by 3D-QSAR studies.

The 3rd example of FP-classification is cpd **7a** and its 2 nearest neighbors **7b** and **7c** (Figure 7). Though the compounds are structurally quite different the values of 2D descriptors for them are very similar and consequently the compounds are assigned to the same class by the classification algorithms.

Commonly most of the data in public compound depositories is compiled from local SAR-studies coming from different research groups. In addition, there is always a cross-laboratory variation in the assay performed, Related to this, the last particular example, 125 nM for **7a** is very close to the chosen threshold (100 nM) for the separation of active and inactive instances in our study and the compound could be active in measurements performed by a different group. Furthermore, at least for the compounds of series **7**, GRIND analysis provides a hypothesis for the misclassifications. Since this 3D QSAR method is alignment independent, compounds with different scaffolds and activity values but sharing the same binding mode could be compared according to the molecular Interaction Field (MIF) maps. The properties of the specific substituents are captured pairwise according to their influence on activity. For example, potential protonation of N in the piperazine (**7b**) and morpholine (**7c**) rings (Figure 7) leads to changes in the molecular interaction fields in comparison to **7a**.

**Figure 8:**
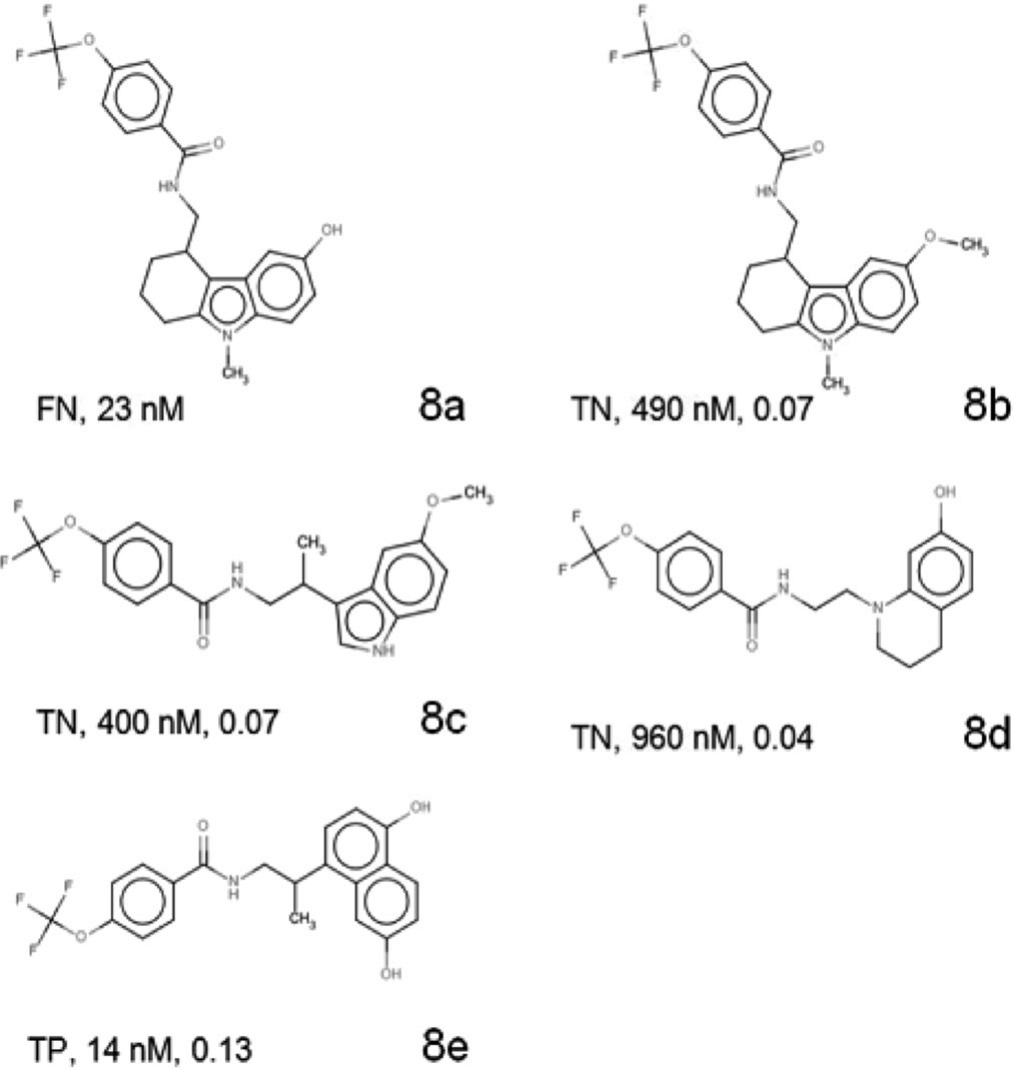
FN-classified compound and its correctly classified nearest neighbors in VSA descriptor space. For each nearest neighbor the Euclidean distance from the misclassified compound in the VSA descriptor space is given.

The last always incorrectly classified compound is **8a** (Figure 8). It was a FN in all overall models and is depicted as a cyan triangle in Figure 4. All five compounds presented in Figure 8 come from one broad SAR-study.^39^ The four neighbors comprise three TN- and one TP-classified compounds. Derivatives **8b** and **8c** share a very similar scaffold with **8a**, the only difference is in the methyloxy substitution of the indole ring which leads to a decrease of potency compared to hydroxyl derivatives. This change in the structure is reflected by slight differences in descriptor values, but could not be captured by the classification algorithms. Additionally, the original SAR indicated that conformational restriction is important for improvement of the activity, e.g. presence of a cyclohexyl moiety in **8d** (960 nM) compared to an indole ring in **8c** (400 nM) and a naphthalene ring in **8e** (14 nM), decreased activity.

The same main trends were observed for VSURF descriptors, although the descriptor values are obtained from 3D MIFs. The respective PCA plot is provided in Figure 4b. Chemical structures and the nearest neighbors of the most prominent outliers are presented on Figures SI-5–8 (Supporting Information).

## 4 Conclusions and Outlook

Recent initiatives for publicly sharing large data sets such as the Open PHACTS initiative (www.openphacts.org)^40^ remarkably increased the chemical space available for building computational models. However, one need to be aware of the fact that data in the public domain are derived from numerous sources and thus vary in quality. In this contribution we chose one target (TRPV1) and systematically analyzed the suitability of a large data set derived from ChEMBL DB for classification studies. By constructing more than 2400 models with different splits into training and test sets, we can conclude that in 99 % of the cases the model built possess an accuracy of more than 60 %.

The analysis of the outliers in the overall models indicated several trends. (1) Minor changes in the structures sharing the same or similar scaffolds could not be captured by the classification algorithms since the descriptor values involved in building the models have very close values for these compounds. (2) The selection of the threshold for assigning active/inactive should be done very carefully and checked during the validation of the model, since removal of false classified instances on the border of the threshold will significantly influence (either improve or decrease the quality) the model. (3) 3D-QSAR, such as GRIND-analysis, could capture these small structural differences and led to satisfactory local models.

Generally, the quality of the obtained classification models strongly depends on the data distribution and the diversity inside the studied data sets. Since these datasets comprise compounds collected from local SAR-studies, data were unevenly distributed, e.g. compounds with similar scaffolds coming from several different studies were overrepresented compared to those investigated in a single study. This has to be considered as a general property of datasets extracted from public sources. Moreover, biological activity measures are derived from functionally different assays, performed in different cell lines. This makes it difficult to compile all data available into one large set and might lead to a drastic reduction of the size of final training set.^41^ Thus, the provenance of the data will be of vital importance for utilizing the full power of open data.

## Supplementary material

As a service to our authors and readers, this journal provides supporting information supplied by the authors. Such materials are peer reviewed and may be re-organized for online delivery, but are not copy-edited or typeset. Technical support issues arising from supporting information (other than missing files) should be addressed to the authors.
