# Supplementary material for: How Far Could We Go with Open Data – A Case Study for TRPV1 Antagonists
Source: Mol Inform. 2013 Jun 18;32(5-6):555–62. doi: 10.1002/minf.201300019 (PMC3743172; doi:10.1002/minf.201300019)
Supplement: Supplementary file 3 [file minf0032-0555-SD3.pdf]

molecular informatics

models – molecules – systems

Supporting Information

© Copyright Wiley-VCH Verlag GmbH & Co. KGaA, 69451 Weinheim, 2013

Equations and Figures.

$$Sensitivity = \frac{TP}{TP+FN} \quad (1)$$

$$Specificity = \frac{TN}{TN+FP} \quad (2)$$

$$Accuracy = \frac{TP+TN}{TP+FP+TN+FN} \quad (3)$$

$$MCC = \frac{(TP*TN)-(FP*FN)}{\sqrt{(TP+FP)(TP+FN)(TN+FP)(TN+FN)}} \quad (4)$$

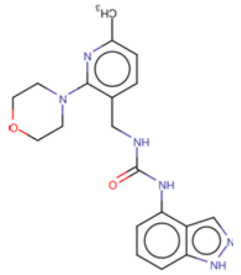

FP, 125 nM

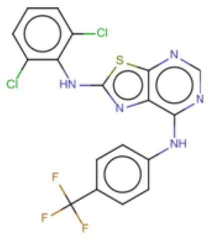

TP, 12 nM

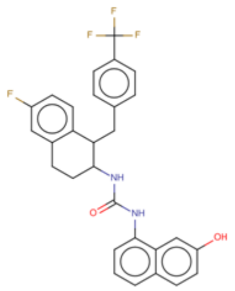

TP, 360 nM

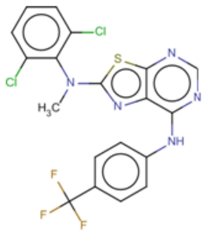

TP, 12 nM

Figure SI-5. FP-classified compound and its TP-classified nearest neighbours in VSURF descriptor space.

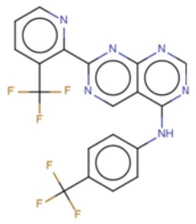

FP, 665 nM

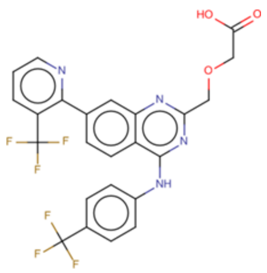

TP, 81 nM

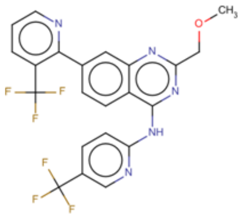

TP, 12 nM

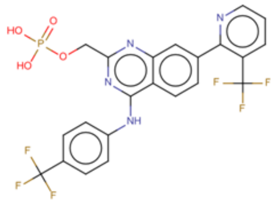

TP, 46 nM

Figure SI-6. FP-classified compound and its TP-classified nearest neighbours in VSURF descriptor space.

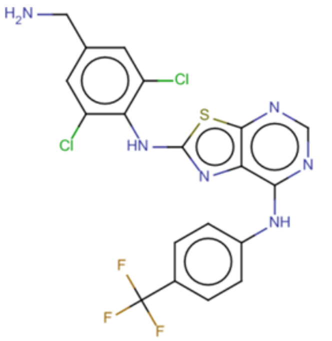

FP, 125 nM

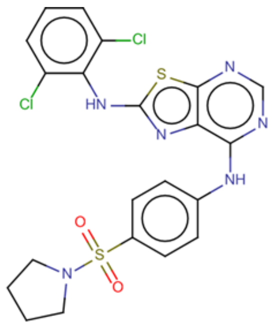

TP, 1 nM

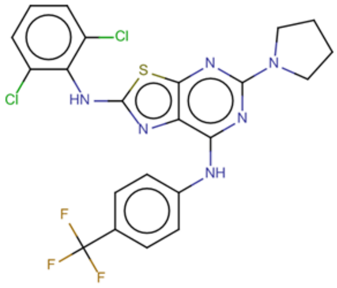

TP, 12 nM

Figure SI-7. FP-classified compound and its TP-classified nearest neighbours in VSURF descriptor space.

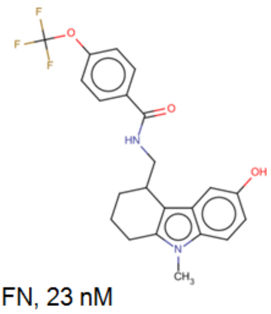

FN, 23 nM

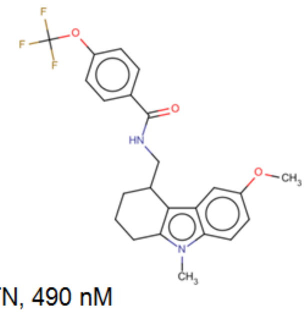

TN, 490 nM

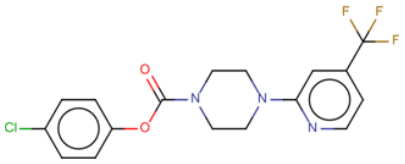

TN, 4600 nM

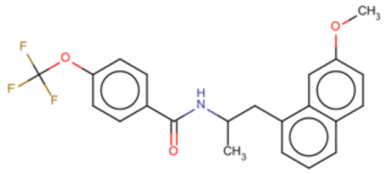

TN, 3100 nM

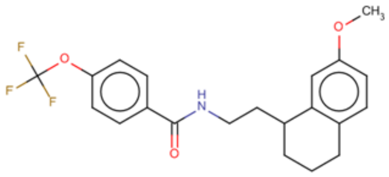

TN, 4650 nM

Figure SI-8. FN-classified compound and its TN-classified nearest neighbours in VSURF descriptor space.
